# Small but mighty: Rhabdomyoblastic melanoma

**DOI:** 10.1016/j.jdcr.2026.01.045

**Published:** 2026-02-03

**Authors:** Surya A. Veerabagu, Kimberly Artounian, Shelly Stepenaskie, Brian Hinds, Andrew Matsumoto

**Affiliations:** a Department of Dermatology, University of New Mexico, Albuquerque, New Mexico; b Department of Dermatology, University of California, San Diego, California

**Keywords:** melanoma with transdifferentiation, rhabdomyosarcomatous differentiation

## Introduction

Rarely, melanoma cells can lose their melanocytic phenotype while simultaneously acquiring nonmelanocytic phenotypes, a process termed transdifferentiation.^1^ It can occur in response to targeted immunotherapy, posing a serious threat to treatment.^2^ It can also occur in the absence of immunotherapy. In both instances, transdifferentiated melanomas are significantly more aggressive than conventional melanoma. Because of the rarity of this phenomenon, no formal treatment guidelines have been established. In this case, melanoma tumor cells transdifferentiated into rhabdomyoblasts, which are early–stage mesenchymal precursors of skeletal muscle cells.

## Case report

An 82-year-old male patient with a history of multiple nonmelanoma skin cancers presented for Mohs consultation for his left superior helix biopsy–proven superficial spreading subtype melanoma in situ with associated dermal pleomorphic malignant melanoma with rhabdomyoblastic features. The initial biopsy was performed by an outside dermatologist, who referred the patient to our medical center. The 2.1 mm-Breslow depth placed the pathologic stage of the tumor at T2b.

**Fig 1:**
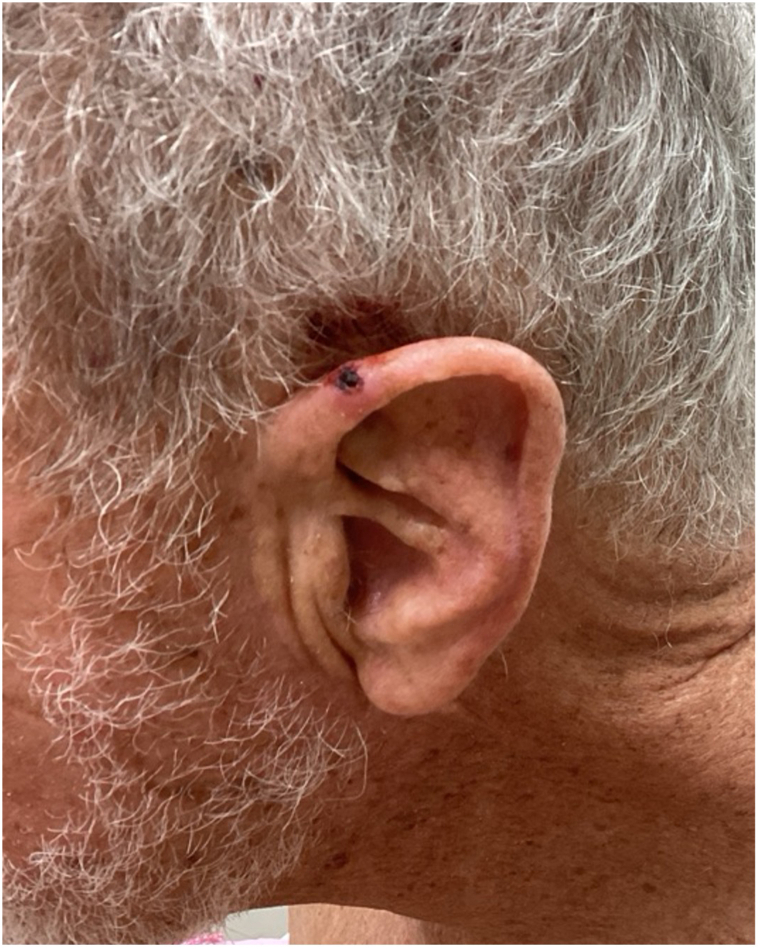
Rhabdomyoblastic melanoma of the ear helix.

Upon our examination, on the left superior helix was a 1.2 cm ulcerated dark brown papule (Fig 1). Physical examination was negative for cervical, parotid, and axillary lymphadenopathy. Positron emission tomography-computed tomography scan was negative for metastatic disease.

**Fig 2:**
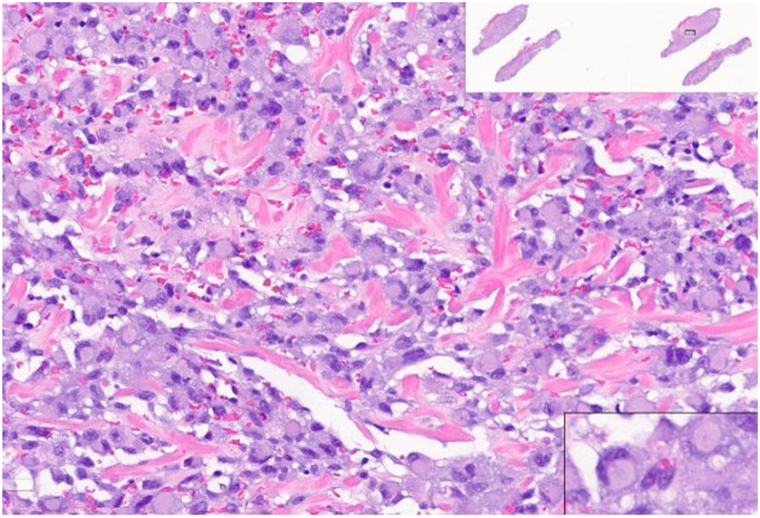
Atypical polygonal cells with prominent cytoplasm and eccentric nucleus.

**Fig 3:**
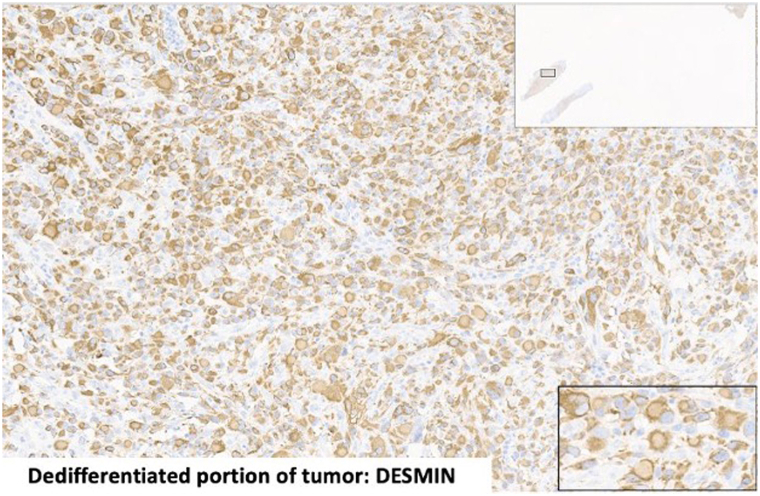
Dedifferentiated tumor portion that was positive for desmin.

Our team performed a confirmatory biopsy. Routine slide evaluation demonstrated a classic superficial spreading subtype melanoma in situ (positive for Sox-10, S100, and PRAME) with an invasive tumor component showing multiple atypical, pleomorphic polygonal tumor cells in the dermis. These atypical, polygonal tumor cells resided within the dermis, without a junctional component (Fig 2). Dermal tumor cells were positive for immunostains Ki-67, PRAME, desmin, myogenin, myoD1, and negative for other melanocytic markers (Sox-10, Melan-A, HMB-45, MiTF, and S100), confirming the diagnosis of an ulcerated, transdifferentiated melanoma with associated rhabdomyosarcomatous phenotype (Fig 3). The malignant dermal cells were negative for common melanoma mutation BRAF V600E. Given the aggressive tumor subtype and Breslow thickness, the patient underwent partial auriculectomy (wide local excision) with sentinel lymph node biopsy performed by head and neck surgery. Lymph node biopsies were negative for tumor. Both the melanoma in situ and invasive transdifferentiated component were excised with clear margins.

After the surgery site healed, the patient successfully completed adjuvant radiation therapy 2 months after surgical excision and is currently undergoing adjuvant pembrolizumab immunotherapy for 1 year. The patient is being closely followed with regular skin checks and regular imaging for monitoring of recurrence or metastatic disease. As of 9 months after surgery, he remains without clinical evidence of recurrence.

## Discussion

Primary cutaneous melanoma with rhabdomyoblastic transdifferentiation is incredibly rare, with only 10 cases confirmed with immunohistochemical evaluation demonstrating loss of melanocytic markers and positivity of both desmin and myogenin.^3^^,^^4^ Patient ages ranged from 41 to 96 with a 7:2 male-to-female ratio. Melanoma subtypes included undisclosed (4/10), superficial spreading (2/10), desmoplastic melanoma (2/10), lentigo maligna melanoma (1/10), and nodular melanoma (1/10) with Breslow depths ranging from 4.1 to 80 mm.^3^ Only 1 of the 10 cases did not include patient outcome information.^5^

The majority (6/10) of cases in the literature were diagnosed with tumor metastases concurrently present. In some cases, patients initially presented to the hospital due to the symptoms from their metastases rather than from the tumors themselves, despite large (>5 cm) clinical sizes.^6^ Of these 6 cases, 6 patients died; however, 1 death was unrelated to cutaneous disease. Three of the 4 melanoma-related deaths occurred, while patients were undergoing various adjuvant therapies, including chemotherapy (dacarbazine), anti-PD1 immunotherapy, and radiotherapy. All 4 patients were male ranging in age from 41 to 76. Notably, patients’ melanoma Breslow depths (4.1-9 mm) were smaller in comparison to other reported cases (4.1-80 mm). Two of the living patients with known metastases were undergoing adjuvant chemotherapy (unnamed agents).

Of the 9 immunohistochemically confirmed cases of primary cutaneous melanoma with rhabdomyosarcomatous transdifferentiation, only 3 cases did not present with metastases, similar to our case.^6^^,^^7^ Two reports only mentioned tumor excision, and it is assumed that neither patient underwent adjuvant therapy. Both cases involved patients in their 80s with facial desmoplastic melanomas (temple and chin) with 18.0 and 80 mm Breslow depths. The third case presented with an associated lentigo maligna melanoma with a Breslow depth of 4.1. However, within 3 months, the tumor recurred twice after excision. After the third excision, the patient underwent adjuvant radiation therapy and remained disease free. All studies, including our own, followed patients for at least 6 months after the original diagnosis.^6^^,^^7^

When reviewing the data, primary cutaneous melanoma with rhabdomyosarcomatous transdifferentiation appears to oppose standard melanoma trends, when reviewing the data-patients with more aggressive melanoma subtypes, such as desmoplastic and lentigo melanoma with larger Breslow depths (18-80 mm), appeared to positively respond to therapy, whereas those with smaller Breslow depths (4.1-9.0 mm) responded poorly. The Breslow depth of these cases ranges from double to 40 times the Breslow depth of our case (2.1 mm).^3^ Furthermore, for most cases, the rhabdomyoblastic de-differentiation was only identified retrospectively after recurrence or nodal metastasis, not at primary presentation like ours.^1^ Whether these are trends or coincidences, these cases emphasize the unpredictability and high mortality of this disease.

Interestingly, rhabdomyoblastic transdifferentiation in primary melanoma cases tends to generally occur later (mean, 70 years old; range, 41-96 years old) and in male patients (4:1 male-to-female ratio) in comparison to rhabdomyoblastic transdifferentiation cases in nodal/distant melanoma metastases. In those cases, patients were significantly younger (mean, 40 years old; range, 21-59) with a female predominance (5:2).^4^^,^^8^

This case not only adds to the literature of this rare diagnosis but also draws attention to the aggressive nature of this tumor subtype that can be mitigated with early, prompt multidisciplinary care. This case also highlights the importance of incorporating extensive, specific dermatopathologic review for consensus pathologic confirmation of these rare diagnoses.

## Conflicts of interest

None disclosed.
